# Ghost introgression facilitates genomic divergence of a sympatric cryptic lineage in *Cycas revoluta*

**DOI:** 10.1002/ece3.10435

**Published:** 2023-08-18

**Authors:** Jui‐Tse Chang, Koh Nakamura, Chien‐Ti Chao, Min‐Xin Luo, Pei‐Chun Liao

**Affiliations:** ^1^ School of Life Science National Taiwan Normal University Taipei Taiwan; ^2^ Botanic Garden, Field Science Center for Northern Biosphere Hokkaido University Sapporo Japan

**Keywords:** archaic introgression, cryptic diversity, cycad, ecological adaptation, sibling species, Tortonian

## Abstract

A cryptic lineage is a genetically diverged but morphologically unrecognized variant of a known species. Clarifying cryptic lineage evolution is essential for quantifying species diversity. In sympatric cryptic lineage divergence compared with allopatric divergence, the forces of divergent selection and mating patterns override geographical isolation. Introgression, by supplying preadapted or neutral standing genetic variations, can promote sympatric cryptic lineage divergence via selection. However, most studies concentrated on extant species introgression, ignoring the genetic legacy of introgression from extinct or unsampled lineages (“ghost introgression”). Cycads are an ideal plant for studying the influence of ghost introgression because of their common interspecific gene flow and past high extinction rate. Here, we utilized reference‐based ddRADseq to clarify the role of ghost introgression in the evolution of a previously identified sympatric cryptic lineage in *Cycas revoluta*. After re‐evaluating the evolutionary independency of cryptic lineages, the group‐wise diverged single‐nucleotide polymorphisms among sympatric and allopatric lineages were compared and functionally annotated. Next, we employed an approximate Bayesian computation method for hypothesis testing to clarify the cryptic lineage evolution and ghost introgression effect. SNPs with the genomic signatures of ghost introgression were further annotated. Our results reconfirmed the evolutionary independency of cryptic lineage among *C. revoluta* and demonstrated that ghost introgression to the noncryptic lineage facilitated their divergence. Gene function related to heat stress and disease resistance implied ecological adaptation of the main extant populations of *C. revoluta*.

## Graphical abstract

Previous population genetics study revealed a sympatric cryptic lineage of *Cycas revoluta* in Taiwan. By detecting the SNP outliers and annotating its functions of lineages sympatric and allopatric to the cryptic lineage, plus approximate Bayesian computation model selection, this article provided a mechanism of the sympatric cryptic lineage divergence by ghost introgression.

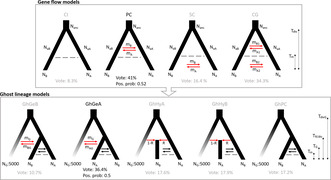

## 1 INTRODUCTION

A cryptic lineage is a genetically distinct group of descendants whose superficial morphologies are indistinguishable from those of a known species. Recognition of this hidden diversity and its ecological role helps us to appreciate the real biodiversity of the world better and formulate workable conservation policies for reducing biodiversity loss arising from ecosystem stresses like climate change (Gomez‐Corrales & Prada, 2020; Hodkinson et al., 2011). Because of the morphogenetic discordance of cryptic lineages, we are interested in how they evolved and how the lineage entities persist genetically (Michalski & Durka, 2015; Monro & Mayo, 2022; Struck et al., 2018).

Compared with allopatric cryptic lineages, which may be mainly influenced by geographical isolation, sympatric cryptic lineages with overlapping population distributions may be caused by divergent selection as explained by Titus et al. (2019), or assortative mating, or allopatric divergence following secondary contact, which was put forward by Binks et al. (2021). Natural selection dominates in the first two mechanisms and will drive adaptive divergence of specific genes, causing genomic islands (Foote, 2018). These genomic islands can have pleiotropic effects (“magic traits”) connected with nonrandom mating that act as barrier loci to promote speciation. Other loci linked to genomic islands could hitchhike to produce genome‐wide divergence. These selection‐driven processes will become efficient under high genetic variations, which can be facilitated by introgression.

Introgression from a third lineage may broaden ecological opportunities (“adaptive introgression”) and confer standing genetic variations, advancing population divergence into new environments (Oziolor et al., 2019; Richards & Martin, 2017) or even facilitating divergence of the introgressed lineage from its sister lineage, making sympatric speciation easier (Richards et al., 2019). A mounting number of studies that focused on introgression between present species, however, ignored the genetic legacy left by extinct (archaic) or unsampled lineages, also known as ghost introgressions (Beerli, 2004). However, we maintain that consideration of ghost introgression is a requisite for correctly inferring the mechanisms of macro‐ and micro‐evolutionary processes (Hahn & Nakhleh, 2016; Herrera‐Alsina et al., 2022; Tricou et al., 2022a, 2022b). By transferring standing genetic variations to introgressed lineages, ghost introgression is another force for accelerating adaptation and speciation (Ottenburghs, 2020). Although ghost introgression is well known for facilitating human colonization and adaptation (Gokcumen, 2020; Racimo et al., 2015, 2017), there has been much less focus on its application to wild plants. This is the first study to explore the genomic divergence of cycads from the perspective of ghost introgression, and *Cycas revoluta* is an outstanding system for investigating the influence of ghost introgression on the formation of sympatric cryptic diversity.

**FIGURE 1:**
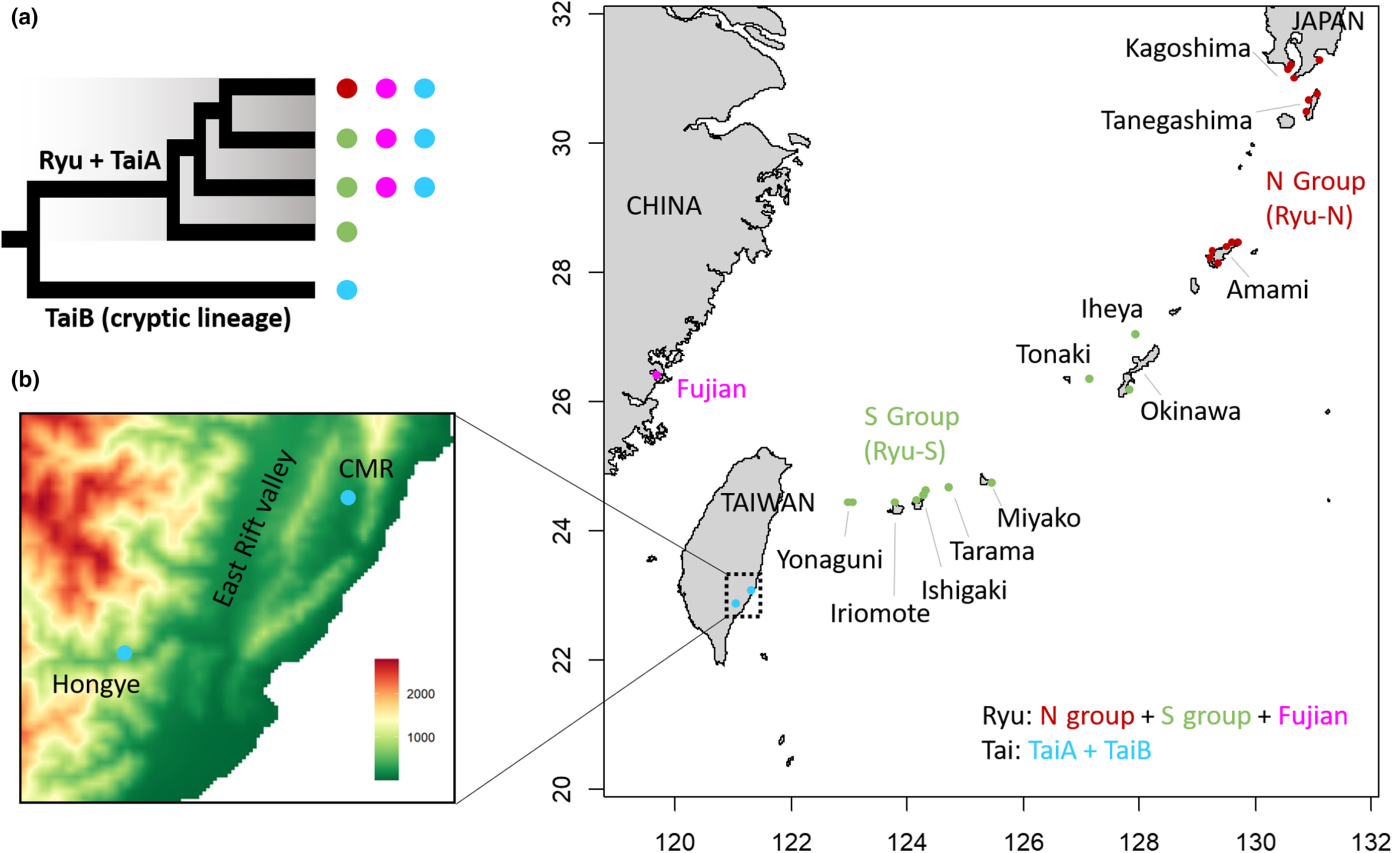
(a) Phylogeny among Ryu + TaiA, and TaiB based on Chang et al. (2022). Colored circles match the classified groups based on geographic and genetic structure. (b) Sampling scheme. See Table S1 for detailed locality and population information. N and S groups are classified as two genetic groups in Chang et al. (2022). TaiA and TaiB refer to two sympatric lineages in CMR and Hongye populations in Taiwan. Ryu group includes Fujian, N, and S groups. CMR, coastal mountain range.

*Cycas revoluta* is mainly distributed along the Ryukyu‐Taiwan Archipelago, and a previous study first found a sympatric cryptic lineage called TaiB, restricted to Taiwan (Figure 1a) with unknown origin and functional divergence (Chang et al., 2022). The main coastal habitats of *C. revoluta* are ecologically different from those of its sister species, *Cycas panzhihuaensis*, from the inland Sichuan basin of China (Liu et al., 2018; Xiao & Moller, 2015), within the area where *Cycas* originated (Liu et al., 2021). The disjunct distribution of *C. revoluta* with long phylogenetic branches and morphological autapomorphy imply a high extinction‐to‐speciation rate during the evolution of *C. revoluta* (Condamine et al., 2015; Liu, Lindstrom, et al., 2022; Walters & Osborne, 2004). The occurrence of multiple extinction events together with frequent hybridization among *Cycas* (Norstog & Nicholls, 1997) makes it worth studying the influence of ghost introgression on sympatric cryptic lineage evolution in *C. revoluta*.

Leveraging the published *C. panzhihuaensis* genome (Liu, Wang, et al., 2022), we realigned the ddRADseq sequences from Chang et al. (2022) (Figure 1b, Table S1). They treated *Cycas taitungensis*, endemic to Taiwan, as a synonym for *C. revoluta* based on overlapping diagnostic traits, paraphyletic relationships, and larger intra‐ than interspecific genetic variations. However, a diverged cryptic lineage restricted to Taiwan was also discovered. According to their genetic and geographical distribution, we separated populations of *C. revoluta* into three groups: Ryu, TaiA, and the cryptic lineage, TaiB (Figure 1a). Group Ryu comprises the Ryukyu and Fujian populations (a small population on the China coast, genetically admixed with the Ryukyu populations); group TaiA is genetically mixed with Ryu, but geographically isolated in Taiwan; and group TaiB is a sympatric cryptic lineage with TaiA in Taiwan, which cannot be distinguished morphologically, but only genetically identified (Figure 1b). By quantifying the genomic divergence among these three groups, we can compare the relative effect of geographical isolation (allopatry) and natural selection (sympatry) on lineage divergence.

In this study, we aimed to (1) scrutinize the natural entity and divergence of TaiB, (2) clarify genomic and functional divergence among allopatric (Ryu‐TaiB and Ryu‐TaiA) and sympatric (TaiA‐TaiB) groups, and (3) test the role of ghost introgression in sympatric divergence (TaiA‐TaiB). Our framework provides a procedure for elucidating the evolutionary background of a sympatric cryptic lineage and highlights ghost introgression as a driver of speciation, particularly for species with low reproductive isolation and high extinction rate.

## 2 MATERIALS AND METHODS

All analyses were performed using packages in R version 3.6.1 (R Core Team, 2019).

### 2.1 Sampling and reference‐based variant calling

The ddRAD raw data were retrieved from Chang et al. (2022). Twenty‐seven populations with 248 individuals, covering the whole distribution range, were examined. Raw reads were de‐multiplexed following PCR duplicates (clone_filter) and phred score <30 reads removal by Stacks 2.53 (Catchen et al., 2013). The reference genome of *C. panzhihuaensis* (Liu, Wang, et al., 2022), a sister species with the same chromosome number (*n* = 11) as *C. revoluta* (Zonneveld, 2011), was used for reference‐based mapping by Bowtie 2 (v2.3.4.1; Langmead & Salzberg, 2012). After coordinate sorting by picard (http://broadinstitute.github.io/picard/, accessed on Feb 18, 2023), the bam files were processed in gstacks and populations programs in Stacks 2.53 for variant calling. Due to genetic admixture and lack of distinct groups, all individuals were treated as one population for the populations program (*p* = 1). Loci were retained if they were present in at least half of the individuals (*r* = .5) with minor allele frequency >5% (‐‐min‐maf = 0.05) and maximum observed heterozygosity <0.8 (‐‐max‐obs‐het = 0.8). For further quality control by vcftools (Danecek et al., 2011), we set the total genotype depth and quality at <3 (‐‐minDP = 3) and 10 (‐‐minGQ = 10), respectively, as missing. Individuals having a missing rate >35% and loci with a missing rate >60% were removed. For more accurate island genetic diversity estimations, the island‐specific missing SNPs were also removed.

Three datasets were created for further analyses: (1) containing all SNPs for detection of group‐wise diverged *F*_ST_ and ghost introgression genetic variations; (2) containing neutral SNPs for quantifying population genetic diversity, population structure, and divergence for the sympatric and allopatric groups; and (3) a dataset containing all SNPs of the Taiwan populations, TaiA and TaiB, for testing the evolution of the cryptic lineage after filtering missing SNP unique to TaiA and TaiB (Table 1). Because of the admixed genetic structure, the genetic outliers were detected and removed by PCAdapt, which detects outliers without predefined grouping (Luu et al., 2017). The optimal *K* in the PCA scree plot was selected by Cattell's rule, and SNPs with −log *p* > 150 were defined as outliers.

**TABLE 1:** Three datasets and their respective analyses and aims.

Dataset	Aims	SNPs	Individuals
Dataset 1: All SNPs	Detecting group‐wise diverged SNPs Detecting ghost introgression signals	938	248
Dataset 2: Neutral SNPs	Calculating summary statistics of sympatric/allopatric groups	860	
Dataset 3: All SNPs of Taiwan	Testing evolution of TaiB	801	

### 2.2 Population structure and divergence

Population structure was analyzed by sparse nonnegative matrix factorization (sNMF) and discriminant analysis of principal components (DAPC) in the LEA (Frichot et al., 2015) and adegenet packages (Jombart, 2008), respectively. In sNMF, the focus was on one global structure combining all individuals, and two separate structures, including Ryu + TaiA and TaiB. The cross‐entropy of serial *K* from 1 to 15 was calculated for the best *K* selection with 1000 iterations. *Q*‐matrices around the optimal *K* were plotted by pophelper (Francis, 2017). In DAPC, we only assessed the population structure of Ryu + TaiA because of the high divergence in TaiB. The best cluster was determined by the lowest BIC via the find.cluster function. The best number of PC for DA was retained by using optim.a.score function.

TaiB divergence was then estimated by an individual neighbor‐joining (NJ) tree and island pairwise *F*_ST_. The NJ tree was reconstructed by Nei's distance with 1000 bootstraps utilizing the aboot function in the poppr package (Kamvar et al., 2014). Pairwise *F*_ST_ was calculated by arlsumstat v3.5.2 (Excoffier & Lischer, 2010). We compared the divergence of TaiB with Ryu + TaiA populations. Hierarchical clustering of *F*_ST_ was also performed via the hclust function from stats after transforming by Euclidean distances.

### 2.3 Population genetic diversity

Island‐based and group‐based (Ryu, TaiA, and TaiB) genetic diversity was calculated by a population program in Stacks 2.53. For estimating observed and expected heterozygosity (*H*_obs_ and *H*_exp_), Wright's inbreeding coefficient (*F*_IS_), and private allele number (*P*_N_) were considered. The private allele frequencies (*P*_F_) of the cryptic, sympatric, and allopatric lineages were also compared.

### 2.4 Group‐wise divergence and functional annotation

In the dataset containing all SNPs (Dataset 1), three kinds of pairwise comparisons were calculated for detecting diverged SNPs between groups: (1) sympatric group with TaiA and TaiB, (2) allopatric group with TaiA and Ryu, and (3) allopatric group with TaiB and Ryu. Significantly diverged SNPs were defined by *p* < .001 in the Fisher's exact test, computed via the populations program (Catchen et al., 2013). A Venn diagram of the three groups was constructed to illustrate the group‐wise divergence patterns.

The corrected AMOVA (analysis of molecular variance) *F*_ST_ was used to compare sympatric and allopatric divergences: (1) *rev* versus *tai*: divergence between *C. revoluta* and *C. taitungensis* from previous taxonomy (TaiB was not included because its cryptic identity was not identified before); (2) Tai versus Ryu: allopatrically driven divergence between Taiwan and Ryu group; (3) TaiB versus Ryu: allopatrically driven divergence of TaiB from Ryu group; and (4) TaiB unique: SNPs that differentiate TaiB from allopatric Ryu and sympatric TaiA. The significantly diverged SNPs were annotated with the closest functional genes within ±1 kbp using *C. panzhihuaensis* as a reference through BEDOPS (Neph et al., 2012) and samtools (Li et al., 2009). Subsequently, the gene sequences were compared with the nonredundant (nr) database by the blastx‐fast method in Blast2GO (Conesa et al., 2005), with the taxonomy range restricted to green plants. The KOBAS‐i (Bu et al., 2021) was implemented for gene annotation against *Arabidopsis thaliana*. For the annotated genes in each group, only gene similarities >60% were kept for gene‐enrichment pathway listing by KEGG, PANTHER, and BioCyc in KOBAS‐i based on *A. thaliana*. Only the enrichments with corrected *p* < .05 were retained.

### 2.5 Evolutionary origin of cryptic lineages

Because of the apparent divergence between TaiB and Ryu + TaiA, we tested their evolutionary dependency by examining the current genetic exchange and the ghost introgression effect by hierarchical model testing using approximate Bayesian computation (ABC) with summary statistics (SumStats) and the random forest method (Pudlo et al., 2016; Raynal et al., 2019). With the ABC model settings, gene flow can be a good indicator for discerning allopatric and sympatric speciation in a species with poor dispersibility, like *C. revoluta*, from a population genetics perspective (Gavrilets, 2003; Richards et al., 2019). Gene‐flow models between TaiA and TaiB were first tested for their evolutionary dependency. However, biased sample sizes between Ryu and TaiB may cause false‐negative detection of gene flow. Thus, we only used the sympatric TaiA, which was genetically admixed with Ryu, to compare with TaiB in SumStats (Dataset 3).

If the two lineages are without current gene flow, complete isolation (CI) and primary contact (PC) would be supported; in contrast, secondary contact (SC) and continuous gene flow (CG) would be preferred. If the two lineages diverged allopatrically, gene flow would stop with the onset of isolation. Thus, CI or SC would be verified; alternatively, PC or CG is favored under sympatric divergence. Subsequently, we tested the ghost lineage models under the best of the four scenarios above. Ghost introgression may confer genetic variations in two ways: (1) gene flow between a hypothetical extinct (“ghost”) lineage and TaiB or TaiA (scenarios GhGeB or GhGeA), or (2) hybridization between the ghost lineage and TaiB (or TaiA) to produce the hybrid lineage TaiA (or TaiB; scenarios GhHyA or GhHyB). The best gene‐flow scenario without ghost gene flow (scenario GhPC) was compared with these four ghost models. The detected ghost introgression should arise from either the extinct species or unsampled extant species, which is now geographically distant from the current distribution of *C. revoluta*. Twenty‐two summary statistics (SumStats) regarding population divergence, expansion, and size (Method S1) were applied in model selection and parameter estimation in the abcrf package (Raynal et al., 2019).

In model selection, the overlap of observed and simulated SumStats was first checked by PCA, then the sufficiency of the reference table size was evaluated by the stability of prior error rate, and posterior probability and models were validated for appropriate distinctions. Lastly, the best model was determined by the most positive votes, the posterior probability, and the goodness‐of‐fit (GOF) test against the observed SumStats. In parameter estimation, the random forest hyperparameters were tuned first to find the proper node size and the number of variables. Second, the sufficiency of the reference table size for parameter estimation was tested for stability by posterior estimation. Lastly, the parameters were estimated using 200,000 simulations. Posterior and prior density distributions were plotted given the observed SumStats. The ABC framework and R script for model selection and parameter estimation were modified from Chapuis et al. (2020) and Dittberner et al. (2022) (prior settings in Tables S2 and S3; framework details in Figure S1 and Method S1).

### 2.6 Ghost genomic introgression signal detection

Genomic signatures of introgressed regions from ghosts tended to show higher linkage disequilibrium (LD) and divergence than extant nonintrogressed regions (Li & Wu, 2021; Ottenburghs, 2020). Several haplotype‐based methods have been developed to statistically search for these signals (e.g., sliding window, haplotype structure with LD pattern, etc.) (Li & Wu, 2021), but they are unsuitable for genomic markers like ddRAD that are based on reduced representation. Consequently, we applied their rationale to identify the candidate introgressed functional genomic regions received from the ghost.

For detecting ghost‐introgressed SNPs, we extracted the significantly diverged TaiB SNPs from Ryu and TaiA, which totaled 395, including 356 and 39 SNPs, respectively (see Section 3). Based on Skov et al. (2018), the lineage receiving a ghost introgression will contain a high density of private SNPs. Therefore, we compared the genomic distribution of private SNPs, *P*_F_, *F*_IS_, and pairwise *F*_ST_ between the ghost‐introgressed and nonintrogressed lineages. As expected, the introgressed regions with the ghost showed clustered private SNPs, strong inbreeding, and genetic divergence with nonintrogressed lineage. If the ghost‐introgressed genes underwent adaptive introgression, then the selective sweep should decrease the heterozygosity with high *P*_F_. Lastly, the candidate SNPs were functionally annotated using the method described above.

## 3 RESULTS

### 3.1 Reference‐based variant calling

**FIGURE 2:**
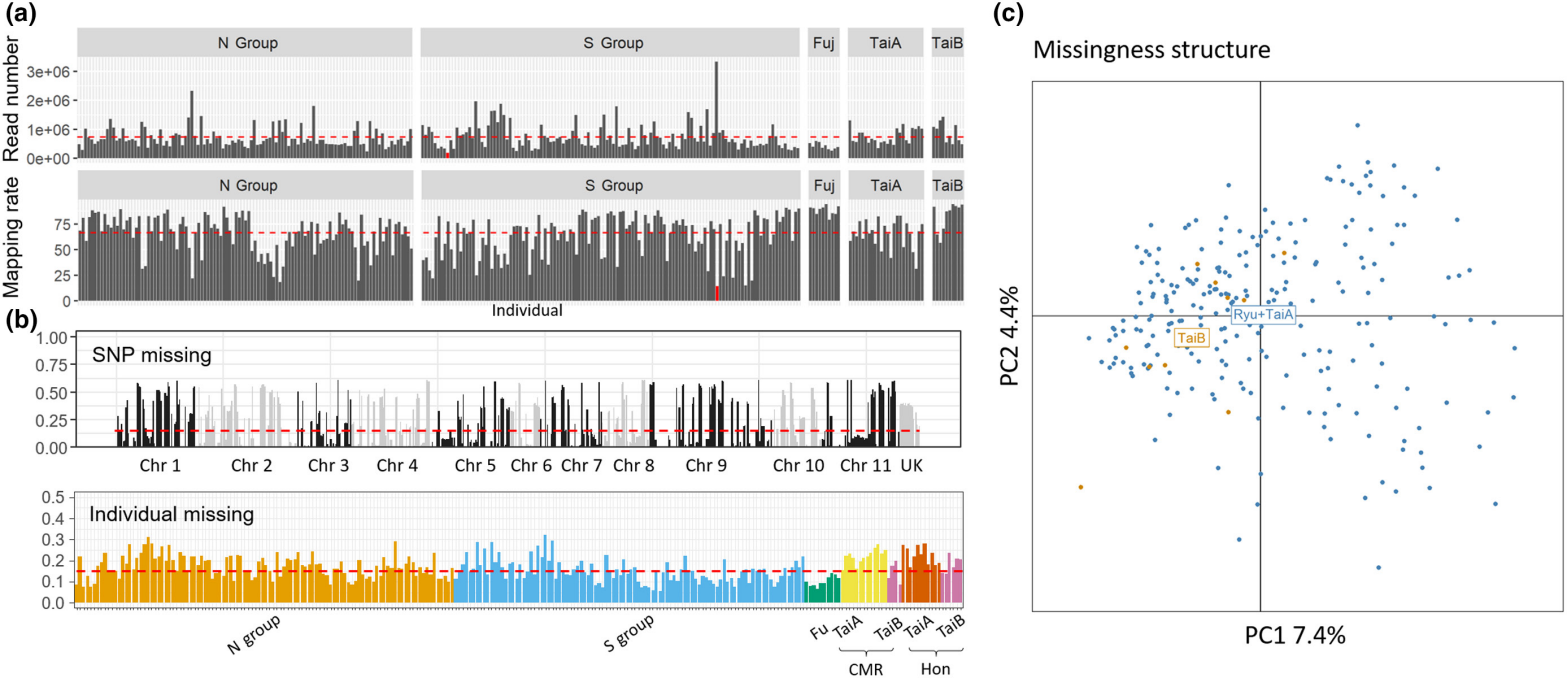
Quality check of raw data and working SNP to confirm the detection of cryptic lineage TaiB. (a) Mapping rate and read number of the raw data, (b) SNP and individual missing rate, and (c) missingness structure of all 938 SNPs. Chr and Fuj refer to chromosome and Fujian population. Red dotted lines refer to the average value in each panel.

After quality control and removal of PCR clones, there were an average of 731,617 reads among 248 individuals. Most individuals had similar read numbers (Figure 2). Mapping the reads against the reference genome resulted in an average mapping rate of 66.05% among 248 individuals (Figure 2). The TaiB and Fujian populations had the highest average mapping rates (83% and 88%, respectively) compared with the Ryukyu Islands and TaiA group (approximately 63%–65%). After variant calling using Stacks, 7266 SNPs were identified. Subsequently, SNP quality control was performed, resulting in the retention of 938 SNPs (83 SNPs across 11 chromosomes on average from Dataset 1) with a missing rate of 17% (Table 1). For Datasets 2 and 3, 860 and 801 SNPs were retained, respectively (Table 1). No individuals were removed from any of the three datasets. The absence of a “missingness” structure (Figure 2) and the intermediate missing rate among cryptic lineage individuals (8%–20% and 5%–32% for all individuals; Figure 2) strongly supported the reliability of the datasets. Moreover, all individuals of cryptic lineage (*n* = 10) came from four different ddRAD libraries, which ruled out the possibility of divergence caused by a batch effect.

### 3.2 Distinct population structure and genetic divergence between Ryu + TaiA and TaiB

**FIGURE 3:**
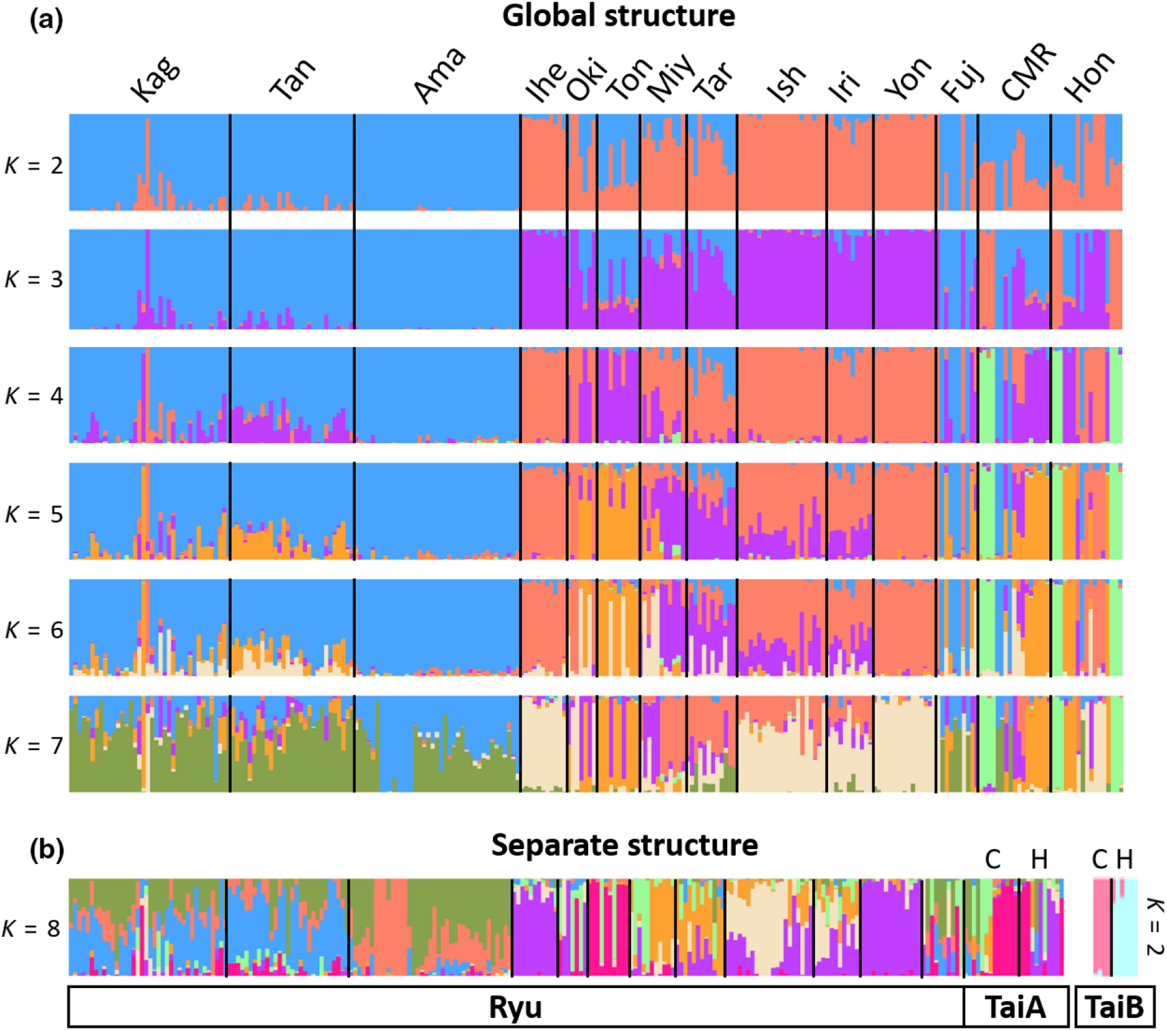
sNMF results of (a) global structure including all individuals and (b) separate structures in Ryu + TaiA and TaiB groups. Only the best *K* of the respective groups were shown in separate structure. C, CMR population; H, Hongye population.

The number of clusters in sNMF was not determined in the global structure because *K* = 2–7 yielded similar cross‐entropy states (Figure S2). The N group in Ryu was separated first (namely Ryu‐N), followed by TaiB from *K* = 3–7 along with the within‐Ryu substructures, Ryu‐N and Ryu‐S (Figure 3a). At the local level, the best clustering was *K* = 8 and 2 for Ryu + TaiA and TaiB, respectively (Figure S2). The former exhibited Ryu‐N/Ryu‐S differences without distinction between the TaiA and Ryu‐S groups, while the latter displayed clear geographic structuring that could even distinguish Hon and CMR of TaiB (Figure 3b). In the Ryu + TaiA cluster, the genetic compositions of Fujian were more similar to those of the Ryu‐N group, while the Amami island was distinct from others in the Ryu‐N, and the genetic composition was diverse among islands for the Ryu‐S, with all genetic components included in TaiA. Discriminant analysis of principal components revealed similar patterns in separating the Ryu‐N and some Fujian individuals from the others (Figure S3).

**FIGURE 4:**
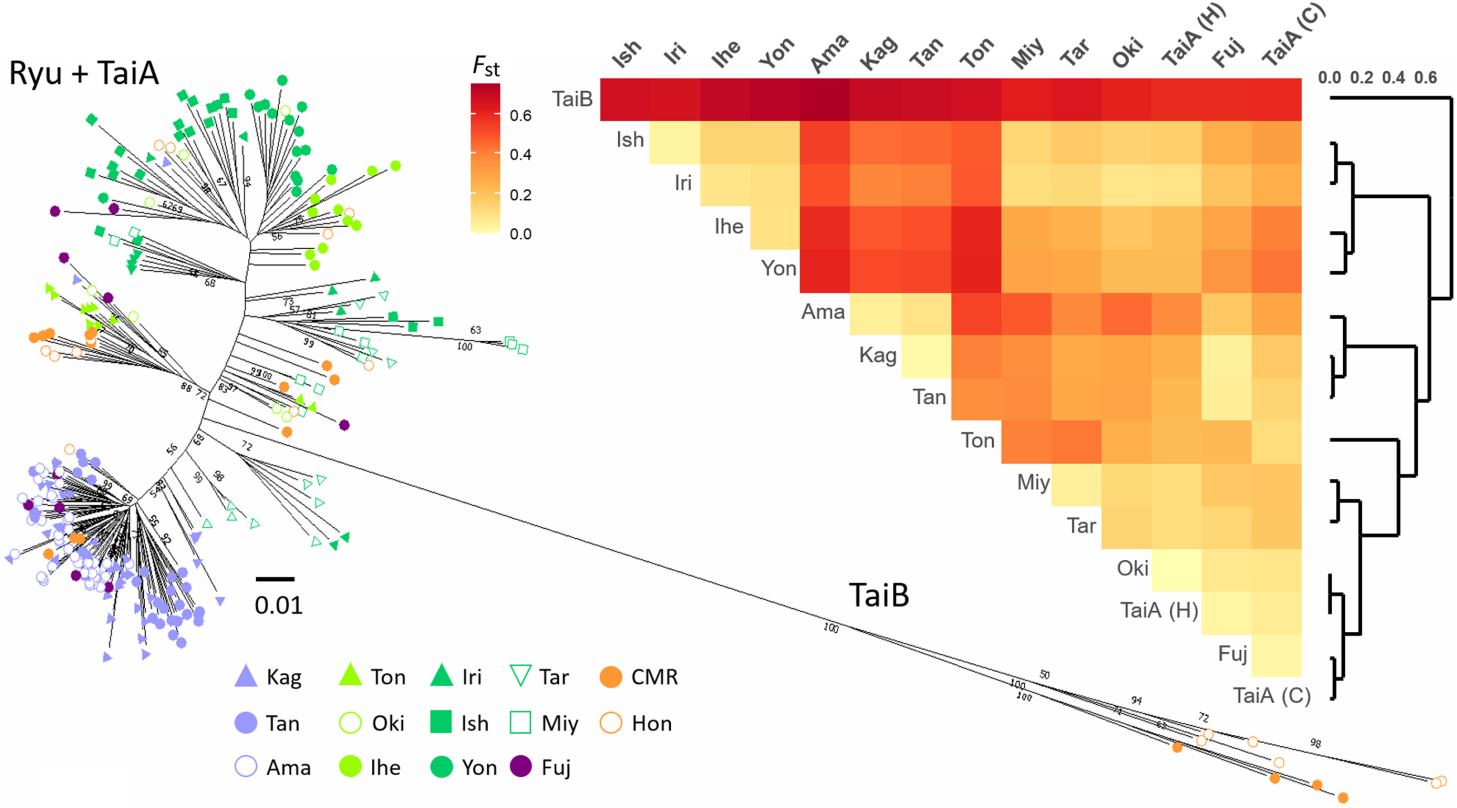
Individual neighbor joining tree and pairwise *F*_ST_ heatmap. Dendrogram beside the heatmap is the hierarchical clustering based on pairwise *F*_ST_ values. Island abbreviations are the first three letters of the names. H and C in parentheses by TaiA represent Hongye and CMR populations, respectively.

Genetic divergence of TaiB was strong, and it was separated from the rest of the individuals and islands in the NJ tree and pairwise *F*_ST_ heatmap, respectively (Figure 4). Ryu and TaiA were in reciprocal paraphyly in the NJ tree, with former individuals clustered in either TaiA group (Figure 4). The divergence within the TaiB clade corresponded to the geographic distribution of Hon and CMR populations, consistent with the population structure.

### 3.3 Retention of ancestral polymorphism despite strong inbreeding in the cryptic lineage

**FIGURE 5:**
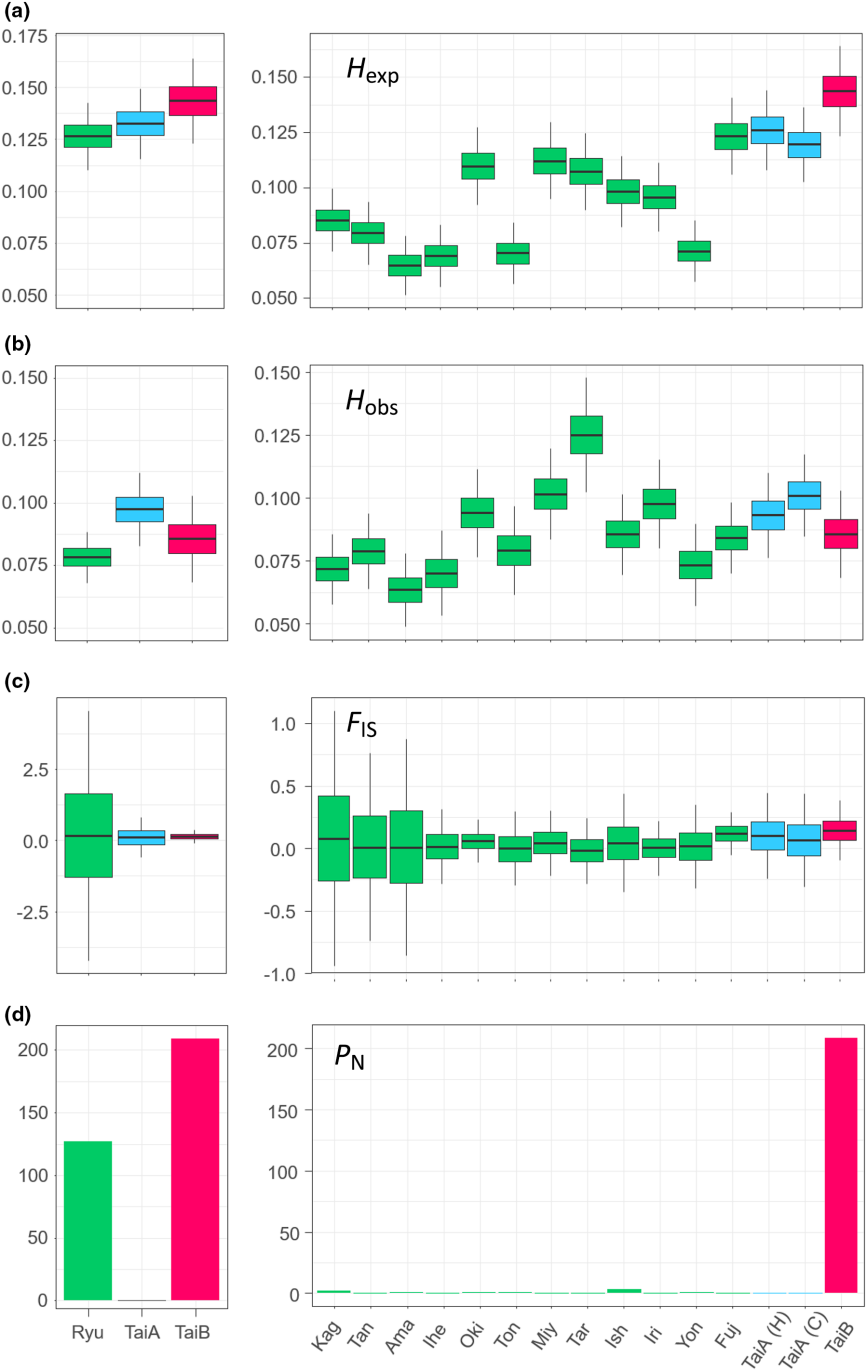
Genetic diversity at group (left) and island level (right). (a) Expected heterozygosity (*H*_exp_), (b) observed heterozygosity (*H*_obs_), (c) inbreeding coefficient (*F*_IS_), and (d) number of private alleles (*P*_N_). Island abbreviations are the first three letters of the names. H and C in parentheses by TaiA represent Hongye and CMR populations, respectively.

*H*_exp_ mainly was higher than *H*_obs_ among groups and islands, except for some small islands such as Yon, Ton, and Tar (Figure 5a), indicating outbreeding of small island populations, probably as sinks from multiple sources. The *H*_obs_ dropped more in TaiB than TaiA, leading to the reverse trend of the two groups between *H*_obs_ and *H*_exp_; Ryu exhibited the lowest value (Figure 5a,b). These differences revealed prevalent inbreeding among the *C. revoluta* populations, as shown by the positive *F*_IS_ (Figure 5c). The inbreeding was strongest in TaiB and most variable in Ryu, with the high variability caused by Ryu‐N (Kag, Tan, and Ama). TaiB exhibited the strongest inbreeding and the highest *P*_N_, which suggested its evolutionary independence from the sympatric TaiA or Ryu (Figure 5d). The high *P*_F_ (mean = 0.368) in TaiB indicates extensive retention of ancestral polymorphisms (Figure S4); contrastingly, the very low *P*_F_ in Ryu + TaiA (mean = 0.036) signifies recent population expansion. The small island populations were nearly panmictic, probably indicating they were sink populations that received genetic variations from multiple sources.

### 3.4 Selective surpassed geographical process for cryptic lineage divergence

**FIGURE 6:**
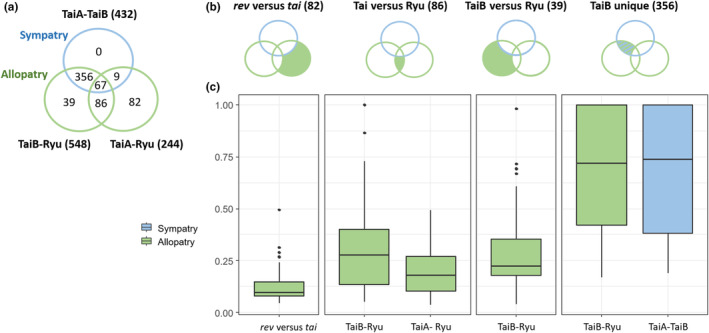
Venn diagram showing number of diverged SNPs among (a) Ryu, TaiA, and TaiB pairwise comparisons, and (b) based on previous taxonomy, Taiwan and Ryu, TaiB and Ryu, and TaiB divergence, in the right order. (c) *F*_ST_ values corresponding to each compared group. Green and blue colors referred to allopatric and sympatric group comparisons, respectively.

Comparisons between groups revealed an abundance of divergent SNPs between Ryu + TaiA and TaiB, with allopatric and sympatric groups (Ryu vs. TaiB, 548, and TaiA vs. TaiB, 432) exceeding Ryu + TaiA (Ryu vs. TaiA, 244; Figure 6a). The Venn diagram shows no unique sympatrically diverged SNPs, and the 432 SNPs in the sympatric group (blue circle, Figure 6a,b) were all shared with the two allopatric groups. In this intersection, Ryu + TaiA versus TaiB contributed 82% (TaiB unique, *n* = 356), corresponding to genome‐wide TaiB divergence. In the three allopatric group comparisons (green filled area, Figure 6b), the number of diverged SNPs driven by allopatry was the highest in Taiwan versus Ryukyu (86), similar to *rev* versus *tai* (82), while the number of unique diverged SNPs in TaiB versus Ryu was the lowest (39). Among these four compared groups, the divergence was strongest, with the average highest *F*_ST_ value in the sympatric group, TaiA‐TaiB, compared with the other three allopatric groups (Figure 6c, Table 2), indicating a stronger selection pressure than geographical processes for TaiB divergence. Notably, the *rev* versus *tai* group exhibited the lowest *F*_ST_ based on the previous taxonomy.

**TABLE 2:** Significantly diverged SNPs between allopatric (Ryu‐TaiB and Ryu‐TaiA) and sympatric pairs (TaiA‐TaiB).

Chromosome	TaiA‐TaiB	Ryu‐TaiB			Ryu‐TaiA		
		N‐B	S‐B	F‐B	N‐A	S‐A	F‐A
1	0.47	0.51	0.49	0.39	0.16	0.05	0.00
2	0.43	0.50	0.54	0.39	0.26	0.15	0.01
3	0.40	0.54	0.46	0.41	0.22	0.16	0.00
4	0.41	0.55	0.49	0.35	0.19	0.19	0.00
5	0.41	0.45	0.44	0.33	0.27	0.15	0.00
6	0.47	0.47	0.44	0.25	0.17	0.22	0.00
7	0.44	0.49	0.51	0.42	0.10	0.15	0.05
8	0.51	0.55	0.53	0.36	0.13	0.15	0.00
9	0.64	0.64	0.60	0.57	0.18	0.15	0.01
10	0.39	0.46	0.54	0.33	0.19	0.28	0.00
11	0.48	0.55	0.50	0.43	0.34	0.11	0.01
Unknown	0.08	0.13	0.13	0.04	0.00	0.04	0.00
Average *F*_ST_ (min–max)	0.63 (0.14–1)	0.61 (0.04–1)	0.64 (0.09–1)	0.69 (0.28–1)	0.15 (0.04–0.57)	0.24 (0.07–0.67)	0.23 (0.18–0.25)
Total outlier sites	432	489	472	374	189	139	7
Total polymorphic site	811	853	910	739	591	604	517
Total site	938						

*Note*: Values in each chromosome are the average *F*_ST_ of SNPs.

Regarding functional annotation, TaiB functionally diverged with Ryu + TaiA mainly in phenology, growth structure, and resistance to abiotic stress and pathogens (TaiB unique, Supporting Information). In contrast to multifunctional divergence, Tai versus Ryu and TaiB versus Ryu were more related to coastal stress and root‐cyanobacterial symbiosis, respectively (Supporting Information). In *rev* versus *tai*, functional divergence was attributed to coastal stress, pathogen resistance, reproductive organ development, and phenology (Supporting Information), suggesting ongoing speciation (Chang et al., 2022).

### 3.5 Ghost introgression and sympatric divergence

Reference table sizes of 70,000 and 100,000 were adequate for model selections, as indicated by converging prior error rates (ranging from 40.1–42.1 and 57.43–57.9 in PC and GhGeA model, respectively) and posterior probability for gene flow and ghost introgression scenarios, respectively. Additionally, the PCA clouds of both model sets covered the observed data with appropriate model validations (54.4% and 68% classification rate for PC and GhGeA model). The GOF test confirmed that the best model fit the observed data (*p* value = .28 and .1 for PC and GhGeA model, respectively) and indicated precise and accurate simulation.

**FIGURE 7:**
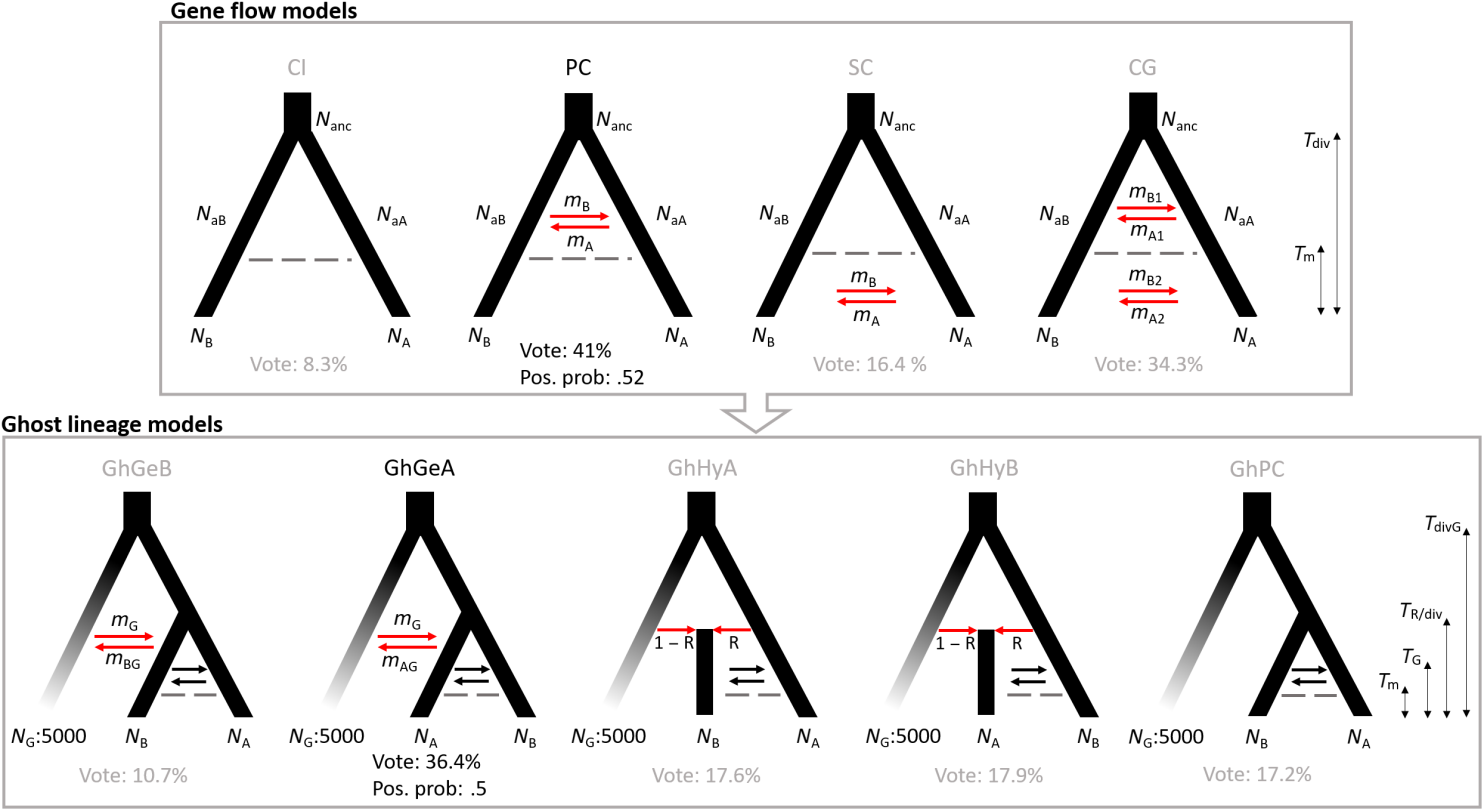
Hierarchical ABC and model selection results. Gray arrows indicate the gene‐flow models as first tested, and the ghost lineage models were then tested based on the best gene‐flow results. Red arrows highlight the differences between models. Parameters are described in Method S1 and results. Pos. prob, posterior probability of the best model. In gene‐flow models: CI, complete isolation; CG, continuous gene flow; PC, primary contact; SC, secondary contact; in Ghost lineage models: GhGeA, ghost gene flow to A; GhGeB, ghost gene flow to B; GhHyA, ghost hybridization with A; GhHyB, ghost hybridization with B; GhPC, no ghost gene flow.

From the model selection of the four gene‐flow scenarios, primary contact outcompeted the others, with a posterior probability of .52 and 41% vote (Figure 7). This suggests that TaiB is an independently evolving lineage under sympatric divergence with Ryu + TaiA. Nested within the primary‐contact model, the scenario of bidirectional gene flow between the ghost and TaiA (i.e., GhGeA) was supported in ghost lineage models, with a posterior probability of .5 and 36.4% vote (Figure 7). Overall, tests of the hierarchical ABC model demonstrated that ghost gene flow to TaiA facilitated the divergence of evolutionarily independent TaiB.

After hyperparametric tuning, a size of 50 nodes and seven variable splits generated the lowest prediction error. Table 3 and Figure S5 show the parameter estimation and posterior distribution for the GhGeA model, respectively. A reference table with 200,000 simulations was sufficient for parameter estimation based on the converged prior error rates. The ancestral *C. revoluta* diverged with the ghost lineage *T*_divG_ generations ago (median = 411,780, mean = 407,920.5; 95% CI = 305,889–496,936). Subsequently, the population declined *N*_anc_ times (median = 2.62, mean = 2.57; 95% CI = 0.1–4.93) to *N*_aA_ (median = 10,318, mean = 10,546.5; 95% CI = 7895–11,623) and *N*_aB_ (median = 11,436, mean = 11,650; 95% CI = 6645–16,904) during the TaiA and TaiB divergence *T*_div_ generations ago (median = 301,758.7, mean = 299,663; 95% CI = 203,376.4–396,386.8) with the emergence of ghost gene flow. The ghost gene flow was twice as high from ghost to TaiA (*m*_G_; median = 4.6 × 10^−4^, mean = 1.3 × 10^−3^; 95% CI = 1.26 × 10^−5^–4.8 × 10^−3^) than in the reverse direction (*m*_A_; median = 2.4 × 10^−4^, mean = 8.3 × 10^−4^; 95% CI = 1.3 × 10^−5^–4.4 × 10^−3^) and lasted for approximately 293,510 generations until the extinction or cessation of gene flow from the ghost lineage *T*_G_ generations ago (median = 8249, mean = 8165.7; 95% CI = 1286–14,726). During the divergence of TaiA and TaiB, primary gene flow was similar between TaiA and TaiB (*m*_A_; median = 1.03 × 10^−3^, mean = 1.76 × 10^−3^; 95% CI = 1.29 × 10^−5^–4.9 × 10^−3^; *m*_B_; median = 1.12 × 10^−3^, mean = 1.73 × 10^−3^; 95% CI = 1.49 × 10^−5^–4.8 × 10^−3^). The gene flow ceased *T*_m_ generations ago (median = 4252, mean = 4336; 95% CI = 3042–5915) together with a bottleneck event, resulting in the population contracting to *N*_B_ (median = 2505, mean = 2442; 95% CI = 1585–2980) and *N*_A_ (median = 2228, mean = 2235; 95% CI = 1525–2969). To eliminate the potential false “ghost” effect caused by the preclusion of Ryu samples, we tested the observed SumStats with all Ryu + TaiA data, and both model selection and parameter estimation were similar to the results above.

**TABLE 3:** Parameter estimation of the best GhGeA model in ABC.

Parameter	Mean	Median	95% CI	post.NMAE.mean^a^
*N*_B_	2441.90	2505	1585–2980	0.15
*N*_A_	2234.90	2228	1525–2969	0.19
*N*_anc_	2.57	2.62	0.1–4.93	2.93
*N*_aB_	11,650	11,436	6645–16,904	0.23
*N*_aA_	10,546.50	10,318	5403–16,737	0.28
*m*_AG_	8.30E‐4	2.40E‐4	1.3E‐5–4.4E‐3	6.64
*m*_G_	1.30E‐3	4.60E‐4	1.3E‐5–4.8E‐3	7.22
*m*_B_	1.73E‐3	1.12E‐3	1.5E‐5–4.8E‐3	6.24
*m*_A_	1.76E‐3	1.03E‐3	1.29E‐5–4.9E‐3	8.02
*T*_m_	4336	4252	3042–5915	0.18
*T*_div_	299,663	301,759	203,376.4–396,387	0.18
*T*_divG_	407,921	411,780	305,889–496,936	0.13
*T*_G_	8165.70	8249	1286–14,726	0.91

^a^ post.NMAE.mean refers to posterior normalized mean absolute error.

### 3.6 Functional annotation implied stress resistance improvement and coastal adaptation of Ryu + TaiA by ghost introgression

Of 395 SNPs diverging in Ryu + TaiA and TaiB, 39 were unique to TaiB, and only four SNPs displayed ghost introgression signals (Table 4). These SNPs were densely localized on chromosome 2 within a 10‐bp region harboring high *P*_F_ (0.52–0.91) and low heterozygosity (0–0.42). High pairwise *F*_ST_ between populations of Ryu + TaiA and TaiB, ranging from 0.17 to 1.0, indicated potential functional divergence. The four SNPs associated with the ghost were part of a 155‐bp feature in the *C. panzhihuaensis* reference with significant similarity (*E*‐value = 1.3 × 10^−26^, 94% similarity) to a protein‐coding gene *HCHIB* in *A. thaliana*. GO terms were associated with five biological processes and two molecular functions (Table 5), suggesting roles in defense against fungi, chitin breakdown, and systematic acquired resistance (SAR), with pleiotropic functions related to heat and drought stress in *Panicum virgatum* grown in semiarid regions, and implied responses to coastal environments (Hayford et al., 2022).

**TABLE 4:** Functionally diverged SNPs between TaiB and Ryu + TaiA with high linkage disequilibrium.

Chromosome	Position	*P*_F_	*H*_o_	*F*_IS_	*F*_ST_TaiB‐TaiA	*F*_ST_TaiB‐Ryu
Chr2	237462871	0.89	0.018	0.904	0.930	0.581
	237462876	0.87	0.019	0.916	0.859	0.570
	237462878	0.91	0.000	1.000	1.000	0.618
	237462879	0.52	0.421	0.160	0.455	0.169

**TABLE 5:** Annotated GO functions of the *HCHIB* gene with ghost introgression signal.

Position	Gene	GO	Related functions
chr2: 237462768–237462922	HCHIB	P: polysaccharide catabolic process P: chitin catabolic process P: cell wall macromolecule catabolic process P: defense response to fungus P: JA and ET systematic resistance F: chitin binding; endochitinase activity	Heat and drought stresses Plant SAR

## 4 DISCUSSION

Previous research synonymized *C. taitungensis* as *C. revoluta* and reported the discovery of a cryptic lineage, TaiB, unique to Taiwan (Chang et al., 2022). In addition to the distinct genetic components and high *F*_ST_ from other islands, the matching of the TaiB population substructure to geography indicated a geographic barrier of the East Rift Valley in eastern Taiwan (Figure 1b), but this barrier did not lead to the divergence of TaiA. The early divergence and absence of current gene flow between Ryu + TaiA and TaiB suggested independent evolutionary trajectories of these two sympatric lineages. Higher group‐wise *F*_ST_ in this sympatric group (TaiA and TaiB) compared with other allopatric groups without in situ diverged SNPs signified that forces other than geographical isolation might be acting on the sympatric cryptic divergence and ghost introgression might play a pivotal role.

### 4.1 Spatiotemporal background for ghost introgression

The best GhGeA model in ABC suggested ancient sympatric divergence of Ryu + TaiA and TaiB, occurring ca. 9 Mya in the late Miocene near the emergence of Taiwan at ca. 5–9 Mya (Sibuet & Hsu, 2004), assuming a generation time of 30 years (Chang et al., 2022). This result implied that the ancestral *C. revoluta* could have originated and diverged into Ryu + TaiA and TaiB in continental East Asia, where *C. panzhihuaensis*, the sister species of *C. revoluta*, was thriving (Liu et al., 2021; Xiao & Moller, 2015). Also, fossils resembling *C. revoluta* and *C. panzhihuaensis* have been discovered in this region (Su et al., 2014). During the late Miocene, there was an end to the global ocean circulation of warm tropical water, which resulted in a shift from a stable, warm climate to a cooler, more seasonal one (Steinthorsdottir et al., 2021). In East Asia, the changes were manifested latitudinally with cooler, drier weather in the northern regions and more seasonal, humid conditions in Indochina and southern East Asia (He et al., 2023), similar to current conditions where *Cycas* species have a high diversity (Lindstrom et al., 2009; Yessoufou et al., 2017). The crown age of the extant *Cycas* also dates back to the late Miocene, with a high diversification rate (Condamine et al., 2015; Liu, Wang, et al., 2022, but also see Liu et al., 2021 for Eocene diversification). These results indicated that the ancient *Cycas* diversity was high in the late Miocene but may have retreated southward to southern East Asia and Indochina with spatial contraction and demographic expansion or remained relatively stable in demography (Xiao & Moller, 2015). The absence of fossil records after the late Miocene in northern East Asia also supports this perspective (Krassilov, 1978; Su et al., 2014; Yokoyama, 1911). Accordingly, as indicated by ABC, the ghost lineage may have been distributed around southern East Asia or Indochina when it went extinct or ceased the gene flow with *C. revoluta* during the middle Pleistocene.

*Cycas* species are known for their superior hybridization ability but are generally inferior in dispersal (Norstog & Nicholls, 1997; Whitelock, 2002). As a result, more condensed species richness and higher abundance of *Cycas* species diversity in southern East Asia and Indochina during the late Miocene, coupled with microspore flow, have enabled prevalent introgression events. *Cycas* are ambophilous, with both wind and insect pollination. Beetles are the primary pollinators for long‐distance dispersal because the wind has a dispersibility of only 2–10 m (Hall & Walter, 2018; Hamada et al., 2015; Kono & Tobe, 2007; Toon et al., 2020; Wang et al., 1997). During the late Miocene, beetle pollinators shifted southward like other insects (Huang et al., 2007; Xu, 2005), maintaining *Cycas* pollination efficiency. Strengthening the East Asia summer monsoon between ~10 and 8.7 Mya may further facilitate pollination during coning periods (He et al., 2023; Hui et al., 2021; Ren et al., 2020). These paleoclimatic and biogeographic shifts of *Cycas* species and their pollinators would have persisted over the long term in Indochina and southern East Asia (Hewitt, 2000; López‐Pujol et al., 2011), supporting the persistent ghost introgression in this area to TaiA. Ghost introgression persisted until the middle Pleistocene ca. 0.24 Mya, during which Taiwan had long (1.55 Mya; Osozawa et al., 2012) been separated from but recurrently connected with the Asian continent by land bridge following glaciation cycles (Kimura, 1996; Nakazawa & Bae, 2018). Due to low dispersibility, ghost and ancestral *C. revoluta* would be distributed para‐ or sympatrically for detectable gene flow, and hence, both ghost and ancestral *C. revoluta* may exist in the Asian continent before colonizing Taiwan by land bridge in the last glacial period (0.1–0.01 Mya). Another possibility is that the ghost lineage would have survived on Taiwan island with *C. revoluta*, indicating higher cycad species diversity than today. Long‐distance migration from the Asian continent to Taiwan would be less possible in a short time (Liu et al., 2021); hence, the scenario for higher cycad species diversity in Taiwan would be more supportive. The cessation of ghost gene flow may reveal extinction under the paleoclimatic/paleogeographic shift, which transited to a period of intensified glaciation. Alternatively, separation and decrease of the distributional range between *C. revoluta* and the ghost lineage may also be a possible explanation, as supported by the isolated current *C. revoluta* population in Taiwan, and recent demographic contractions observed in many *Cycas* species (Feng et al., 2014; Gong et al., 2015; Liu et al., 2015; Zhan et al., 2011; Zheng et al., 2016). In summary, the GhGeA model in ABC suggests that the significant influence of ghost introgression on Ryu + TaiA and TaiB divergence since the late Miocene can be attributed to the paleoclimate‐driven spatial dynamics of *Cycas* populations.

### 4.2 Ghost introgression facilitates sympatric cryptic lineage divergence and ecological adaptation

Ghost introgression could facilitate adaptation and speciation by transferring advantageous genetic variations to its receivers. The persistence of ghost introgression for ca. 8 million years has provided Ryu + TaiA with a rich source of adaptive genetic variations, potentially facilitating exaptation or local adaptation under novel environments. The colonization of *C. revoluta* in the Taiwan‐Ryukyu Archipelago likely occurred through either a land bridge or when these islands were part of the Eurasian continental arc (Jiang et al., 2019; Li et al., 2017; Osozawa et al., 2012). Both eastward colonization routes exposed the populations to novel coastal environments from continent to island. Given the similarly large effective population size in Ryu + TaiA and TaiB, natural selection from the new coastal environment was likely to have been efficient for both lineages and accelerated their adaptive divergence.

Ghost introgression from the coastal‐adapted lineage may have broadened the ecological resource spectrum of Ryu + TaiA and facilitated ecological adaptation from the ancestral inland to the coastal site (Germain et al., 2021). Spatial expansion may occur on these islands (Chiang et al., 2009). Although the nature of a reduced representation approach, such as ddRADseq, limits the detection of the ghost introgression signal, one functionally diverged candidate gene, HCHIB, related to ghost introgression was found. HCHIB primarily functions as ethylene‐ and jasmonic acid‐mediated SAR against fungus diseases, such as blister blight (de Queiroz et al., 2020; Pathak et al., 2020; Zhang et al., 2022).

Blister blight is a common worldwide problem for growers of *C. revoluta* (Faraz et al., 2020; Weber, 1944), with the symptom severity being positively associated with manganese (Mn) deficiency (Dehgan et al., 1994). The Mn deficiency is often linked to limestone areas (Kavvadias & Miller, 1999; Kowalenko & Ihnat, 2010), the primary habitats of the Ryukyu populations found in coastal regions. Furthermore, coastal environments commonly experienced drought and heat stress, which were found to be associated with pleiotropic functions of HCHIB (Hayford et al., 2022), making populations with introgressed HCHIB better adapted to coastal habitats. Apart from HCHIB, other functionally diverged genes between Ryu + TaiA and TaiB have been identified across the genome, except for chromosome 6 (Table S4). This functional genomic divergence is related to phenology, vegetative and reproductive growth (Supporting Information). While it remains unclear whether they directly drive or maintain sympatric divergence, functional genomic divergence coupled with ghost introgression may not only be related to temporal reproductive isolation during different coning periods but also reshaping the adaptive landscape and facilitating reproductive isolation by reducing gene flow or hybrid breakdown (McGirr & Martin, 2020; Richards et al., 2019). To verify this hypothesis, further studies on morphology and phenology with the interaction with microhabitats in Taiwan are needed (Scriven et al., 2016). The bottleneck event in the late Pleistocene may also have contributed to the reproductive isolation between Ryu + TaiA and TaiB.

TaiB lacks ghost genetic variations compared with Ryu + TaiA, indicating a narrower ecological resource spectrum and less functional genomic evolvability. Thus, TaiB may be susceptible to population contraction or limited geographical range when encountering strong natural selection from a novel environment such as the coastal region. These demographic processes may contribute to the current small population restricted in Taiwan, genetically converted the shared standing genetic variations to private ones during the extirpation, and increased the private allele number and frequency in the remaining populations. This relic ancestral genomic signature of the current TaiB population is supported by the most abundant neutral *P*_N_ (*N* = 209) and highest *P*_F_ (*Q*1–*Q*3 range from 0.4 to 0.94, Figure S4). The absence of distinctive divergent SNPs between sympatric TaiA and TaiB (*blue circles*, Figure 6) also suggests that the divergence was not driven by the in situ emergence of newly derived SNPs. The significant drop in *H*_obs_ (mean = 0.086) from *H*_exp_ (mean = 0.14) among the three lineages raised concerns about severe inbreeding in TaiB (Figure 5a,b). Compared with the Ryukyu Archipelago and southern Kyushu, the habitat of *C. revoluta* in Taiwan was stable and more similar to its ancestral area in continental East Asia (Tsukada, 1966), serving as a refugium for preserving relic TaiB. However, for the Ryu + TaiA group, which would have an ecological shift from the continent ancestral populations, the Taiwan habitat could be a suboptimal niche for the marginal TaiA population, resulting in a similar inbreeding level but a lower private allele number with TaiB.

### 4.3 Conservation of the invisibilities: cryptic lineage and ghost introgression

The recognition of cryptic lineages is important for accurate biodiversity estimation and effective conservation planning (Delic et al., 2017; Oury et al., 2021). However, caution must be exercised when dealing with recently derived cryptic lineages that lack reproductive isolation, as gene flow may hamper the detection of cryptic diversity and lead to taxonomic inflation (Chan et al., 2020, 2021). Also, artificial genetic pollution from its congeners should be avoided. Contrarily, for the ancient divergent cryptic lineages with relic ancestral polymorphisms, such as TaiB of *C. revoluta*, protection of their refugial habitats is imperative. In Taiwan, the woody flora was comprised of paleo‐ and neo‐endemics, forming a “mixed endemism” with high phylogenetic diversity and conservation value (López‐Pujol et al., 2011; Wang et al., 2022). Correspondingly, the coexistence of relic TaiB and coast‐adapted TaiA in sympatry highlights the importance of conservation efforts in southern Taiwan (Cheng et al., 2005; Chiang et al., 2012; Huang et al., 2002; Kuo et al., 2009; Su et al., 2016; Wu et al., 2006). Compared with TaiB, the flourishing Ryu + TaiA around the coast may have been introgressed by ghosts carrying the *HCHIB* resistance gene. Therefore, the study of ghost introgression can shed light on ancient ecological roles and inform conservation paleobiology (Dillon et al., 2022). Future research should integrate functional genomics, ancestral area reconstruction, and geohistorical and paleoecological data to enhance the beneficial effects of conservation efforts.

## 5 CONCLUSIONS

The present study utilized reference‐based ddRADseq to investigate the nature of sympatric cryptic lineages and evolution in *C. revoluta*. Our results revealed that ghost introgression was crucial in explaining sympatric divergence. Functional divergence of candidate genes from archaic gene flow implied adaptive introgression, facilitating ecological adaptation of Ryu + TaiA among islands from its continental ancestry. Moreover, the bottom‐up functional annotations illuminated possible physiological and phenological differentiation, while top‐down studies on the inconsistent morphological and ecological shift between Ryu + TaiA and TaiB can benefit taxonomic treatment and morphological evolution. Without careful morphological examination, early cryptic divergence in *C. revoluta* could have indicated morphological stasis following stabilizing selection, morphological convergence, or pseudo‐cryptic divergence.

Overall, our findings shed light on the importance of ghost gene flow on extant species' adaptation and genomic divergence. The ghost would be the ancient cycad species or the unsampled species whose current distribution is distant from the focal species. While studies of genetic signatures and consequences of archaic introgression focus on humans due to their accessible archaic genome, we urge more attention to archaic introgression in nonmodel plants, particularly for those with less reproductive isolation than animals, to unravel the intricate web of life.

## AUTHOR CONTRIBUTIONS

**Jui‐Tse Chang:** Conceptualization (equal); formal analysis (equal); writing – original draft (equal); writing – review and editing (equal). **Koh Nakamura:** Writing – review and editing (equal). **Chien‐Ti Chao:** Writing – review and editing (equal). **Min‐Xin Luo:** Writing – review and editing (equal). **Pei‐Chun Liao:** Conceptualization (equal); funding acquisition (equal); resources (equal); supervision (equal); writing – review and editing (equal).

## Supporting information


Appendix S1

## Data Availability

Variant call format of three datasets and annotated genes among sympatric and allopatric groups comparisons were deposited in FigShare: https://figshare.com/projects/Ghost_introgression_facilitates_genomic_divergence_of_a_sympatric_cryptic_lineage_in_Cycas_revoluta/166808.
