# Supplementary material for: Ghost introgression facilitates genomic divergence of a sympatric cryptic lineage in Cycas revoluta
Source: Ecol Evol. 2023 Aug 18;13(8):e10435. doi: 10.1002/ece3.10435 (PMC10439367; doi:10.1002/ece3.10435)
Supplement: Supplementary file 1 — Appendix S1 [file ECE3-13-e10435-s001.docx]

**Ghost introgression facilitates genomic divergence of a sympatric cryptic lineage in *Cycas revoluta***

**Running title:** **Ghost introgression in *Cycas***

Jui-Tse Chang^1^, Koh Nakamura^2^, Chien-Ti Chao^1^, Min-Xin Luo^1^, Pei-Chun Liao^1,^*

1. School of Life Science, National Taiwan Normal University, Taipei 11677, Taiwan

2. Botanic Garden, Field Science Center for Northern Biosphere, Hokkaido University, Sapporo 060-0003, Japan

* Corresponding author: Dr. Pei-Chun Liao

Tel: +886-2-77346330

Fax: +886-2-29312904

Email: [pcliao@ntnu.edu.tw](mailto:pcliao@ntnu.edu.tw)

Email addresses of authors:

Jui-Tse Chang: [b03612004@ntu.edu.tw](mailto:b03612004@ntu.edu.tw)

Chien-Ti Chao: [ff8bahamut@gmail.com](mailto:ff8bahamut@gmail.com)

Koh Nakamura: [kohnakamur@gmail.com](about:blank)

Min-Xin Luo: [xji6aup3vup@gmail.com](mailto:xji6aup3vup@gmail.com)

ORIGINAL ARTICLE

Method S1 ABC framework by random forest

In gene flow models, the effective population size (Ne) and gene flow (m) of TaiA and TaiB were set refer to Chang et al. (2022). Due to small census population size in Taiwan, smaller Ne ranging from 10-3,000 was considered. Also, relative population size was limited to bottleneck scenario with 1.01-10 times population contraction. Because of poor dispersability in *C. revoluta*, demography change is likely to connect with gene flow. Hence, time of relative population size change was coupled with gene flow change. Based on the divergence time estimation of TaiB with Ryu+TaiA in Chang et al. (2022) (ca. 10^5^ generation ago considering substitution rate 9.87$\times$10^-8^ from weighted median of ABC and 0.01 substitution in TaiB divergence from BEAST estimation), the maximum value of TaiB and Ryu+TaiA divergence (Tdiv) together with gene flow change (Tm) were set to 7.85$\times$10^5^, with the rule of Tm < Tdiv. Parameter prior distribution with min-max range larger than 3 orders were accounted to be log-uniform. 22 SumStats including total, mean, standard deviation (SD), population, and population specific SD heterozygosity; total, mean, SD, and population segregating site; population private allele; mean, SD, and population pairwise difference (π); *F*is; *F*it; population pairs of *F*st and π. Collinearity will be tackled in the random forest processes and hence not examined.

After PCA check for observed data within simulation data, model selection was tested by 10,000, 20,000, 30,000, 50,000, and 70,000 reference table size for assuring enough simulations by converged prior error rate and posterior probability of the best model. With appropriate model validation, the largest reference table with 70,000 simulations was used for formal model selection. The fit of observed data to the best PC model was then confirmed by GOF. Due to unexpected ghost gene flow effect on parameter estimations (Beerli, 2004; Slatkin, 2005; Tricou, Tannier, & de Vienne, 2022a, 2022b), all parameters were estimated in ghost lineage model, and hence only model selection was performed in the gene flow models.

For the nested ghost lineage model, to decrease the model complexity, we fix the ghost effective population size (N_G_) as 5,000. Gene flow from ghost was set as the same range between TaiA and TaiB (i.e., 10^-5^-0.005). Based on estimated stem age of sect. *Asiorientales* (Liu, Lindstrom, Marler, & Gong, 2021), ghost divergence time was set between 3$\times$10^5^-5$\times$10^5^ generations. Assuming ghost gene flow start after TaiB and TaiA divergence, the cessation of ghost lineage gene flow (T_G_) was set from 1,000-15,000 with the rule of T_div_ < T_divG_. Identical model selection processes except for 1,500 regression trees as gene flow models was performed. After hyperparameter tuning, we first used 100,000 simulations with 1,000 trees to estimate all parameters, then the parameter prior range was narrowed down to be more precisive for the second run based on the results. Reference table size with 100,000, 150,000, and 200,000 simulations were tested for stable posterior estimates. 200,000 simulations with 2,000 trees were applied for final parameter estimations. The graphical framework is shown in Fig. S1.

Table S1 Details of population samplings for ddRADseq.

| **Biogeographic region** | **Locality** | **Sampled Population** | **N for ddRAD** |
| --- | --- | --- | --- |
| Eurasian mainland | Fujian | Fuj | 10 |
| Northern Ryukyu | Kagoshima | Kag 25 | 8 |
|  |  | Kag 26 | 8 |
|  |  | Kag 27 | 12 |
|  |  | Kag 28 | 10 |
|  | Tanegashima | Tan 21 | 10 |
|  |  | Tan 23 | 9 |
|  |  | Tan 24 | 11 |
| Central Ryukyu | Amami | Ama 1 | 8 |
|  |  | Ama 2 | 6 |
|  |  | Ama 3 | 6 |
|  |  | Ama 4 | 6 |
|  |  | Ama 5 | 5 |
|  |  | Ama 6 | 8 |
|  | Iheya | Ihe | 11 |
|  | Okinawa | OkiN | 1 |
|  |  | OkiS | 6 |
|  | Tonaki | Ton | 10 |
| Southern Ryukyu | Miyako | Miy | 11 |
|  | Tarama | Tar | 12 |
|  | Ishigaki | Ish 5 | 7 |
|  |  | Ish 6 | 7 |
|  |  | Ish 7 | 7 |
|  | Iriomote | Iri | 11 |
|  | Yonaguni | Yon 2 | 8 |
|  |  | Yon 4 | 7 |
| Taiwan | Coastal Mountain Range | CMR | 17 (4 TaiB, 13 TaiA) |
|  | Hongye | Hon | 13 (6 TaiB, 7 TaiA) |

Table S2 Prior setting of the PC model.

| **Parameter setting** | |  |  |  |
| --- | --- | --- | --- | --- |
| //all N are in number of haploid individuals | | | |  |
| //#isInt? | #name | #dist. | #min | #max |
| 1 | NeB | unif | 10 | 3000 |
| 1 | NeA | unif | 10 | 5000 |
| 0 | relB | unif | 1.01 | 10 |
| 0 | relA | unif | 1.01 | 10 |
| 0 | Nanc | logunif | 0.01 | 10 |
| 0 | mB | logunif | 1E-9 | 0.1 |
| 0 | mA | logunif | 1E-9 | 0.1 |
| 1 | Tm | logunif | 5 | 7.85E+5 |
| 1 | Tdiv | unif | 1E+3 | 7.85E+5 |
| **Parameter rules** | |  |  |  |
| Tm < Tdiv | |  |  |  |
| **Complex parameter** | | |  |  |
| 1 ancB = NeB*relB output | | |  |  |
| 1 ancA = NeA*relA output | | |  |  |

Table S3 Prior setting of the GhGeA model after prior range adjustment.

| **Parameter setting** | | |  |  |  |  |
| --- | --- | --- | --- | --- | --- | --- |
| //all N are in number of haploid individuals | | |  |  |  |  |
| //#isInt? | #name | #dist. | #min | | | #max |
| 1 | NeB | unif | 1500 | | | 3000 |
| 1 | NeA | unif | 1500 | | | 3000 |
| 0 | relB | unif | 3 | | | 6 |
| 0 | relA | unif | 3 | | | 6 |
| 0 | Nanc | unif | 0.01 | | | 5 |
| 0 | mAG | logunif | 1E-5 | | | 0.005 |
| 0 | mG | logunif | 1E-5 | | | 0.005 |
| 0 | mB | logunif | 1E-5 | | | 0.005 |
| 0 | mA | logunif | 1E-5 | | | 0.005 |
| 1 | Tm | unif | 3000 | | | 6000 |
| 1 | Tdiv | unif | 2E+5 | | | 4E+5 |
| 1 | TdivG | unif | 3E+5 | | | 5E+5 |
| 1 | T_G_ | unif | 1000 | | | 15000 |
| **Parameters rules** | |  |  | | |  |
| Tdiv < TdivG | |  |  | | |  |
| **Complex parameter** | | |  |  |  |  |
| 1 ancCL = Ne_CL*relCL output | | |  |  |  |  |
| 1 ancNCL = Ne_NonCL*relNCL output | | |  |  |  |  |

Table S4 Annotated genes across genome of the significantly diverged SNPs in TaiB and Ryu+TaiA comparisons.

| **SeqName** | **Positions** | **Gene name** |
| --- | --- | --- |
| chr1 | 1113618485-1113619785 | TCP2 |
| chr2 | 804833425-804834988 | AMT1;1 |
|  | 1062760265-1062760817 | FATB |
|  | 237462768-237462922 | HCHIB |
|  | 1034104158-1034106065 | MTHSC70-2 |
|  | 836663379-836664104 | OPR3 |
|  | 1151468400-1151468790 | RIPK |
|  | 1248093314-1248095888 | SCR |
|  | 1217385938-1217386803 | TBL16 |
| chr3 | 611570189-611571773 | CYP94D2 |
|  | 761023359-761024664 | GAE3 |
|  | 404561863-404586760 | PAP27 |
| chr4 | 550519258-550529834 | DCAF1 |
|  | 416196445-416200935 | RBOH_F |
| chr5 | 176404637-176410840 | BIG |
| chr7 | 755526336-755526667 | AVP1 |
|  | 603628717-603630454 | CSLD2 |
|  | 362449808-362450615 | HB21 |
|  | 528908089-528908279 | NAC080 |
| chr8 | 305878713-305881336 | HSL1 |
|  | 403997272-403997697 | iPGAM1 |
|  | 220338859-220339345 | MLO2 |
|  | 481592001-481592426 | SLO2 |
|  | 730398018-730402476 | TMN7 |
| chr9 | 644944442-644946764 | ARA12 |
|  | 524407526-524407712 | CalS7 |
|  | 666639962-666642997 | CYP78A7 |
|  | 252038213-252038833 | D6PKL2 |
|  | 250397287-250399234 | ERF-1 |
|  | 637889643-637889919 | F3H |
|  | 137085010-137085152 | GASA6 |
|  | 116330661-116333203 | HSL1 |
|  | 180237835-180240580 | LHCA3 |
|  | 67051641-67134924 | OXP1 |
|  | 509427483-509427857 | RL6 |
|  | 604445380-604445750 | TAF2 |
|  | 573062551-573063736 | UFO |
| chr10 | 714380455-714462997 | ftsh4 |
|  | 115143241-115143457 | PMDH1 |
| chr11 | 472120585-472121031 | APK2B |
|  | 456987595-456987928 | bHLH39 |
|  | 133758203-133758398 | NIP5;1 |
|  | 67865691-67867281 | SHR |


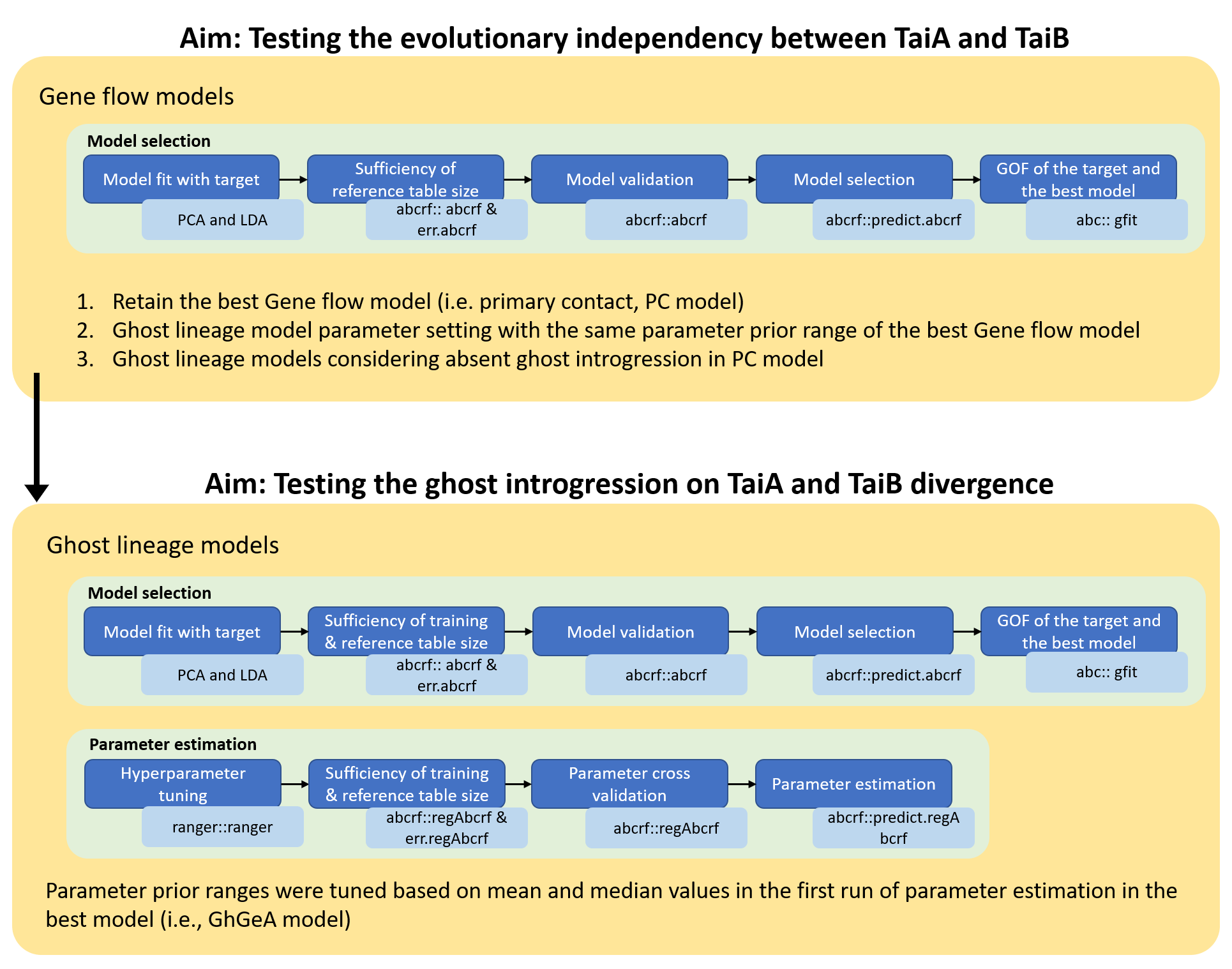


Fig.S1 ABC framework for hierarchical model selections.


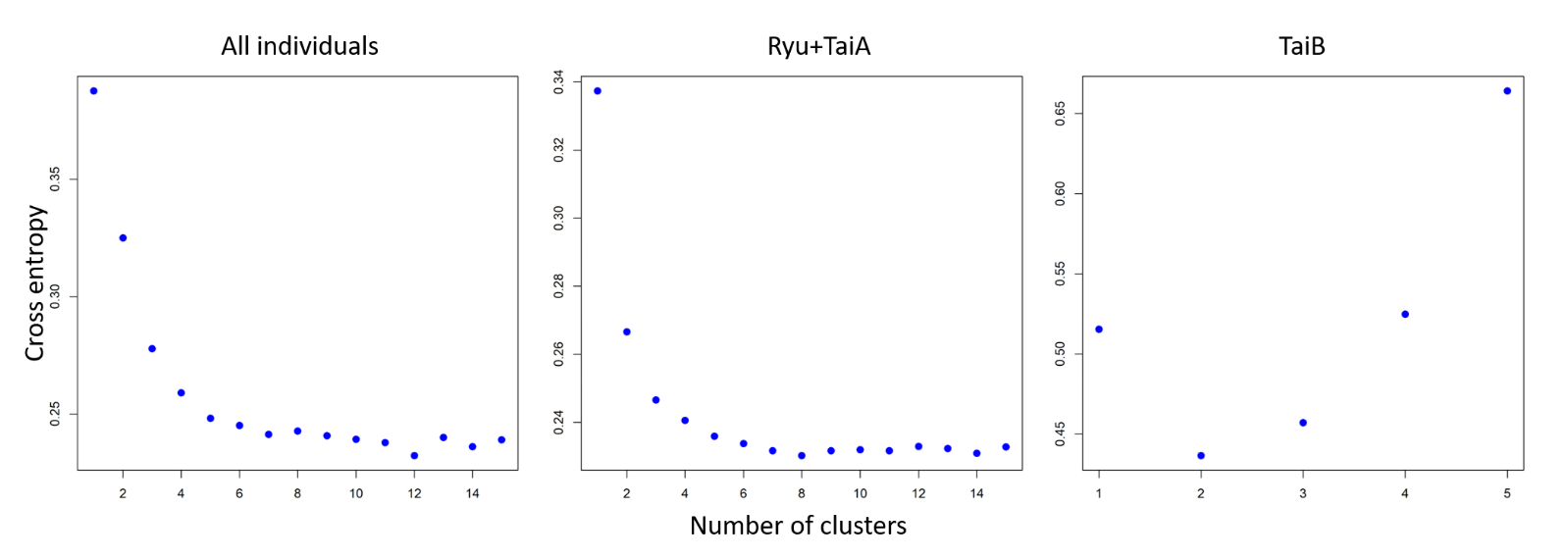


Figure S2 Cross entropy of all individuals, Ryu+TaiA, and TaiB for sNMF.


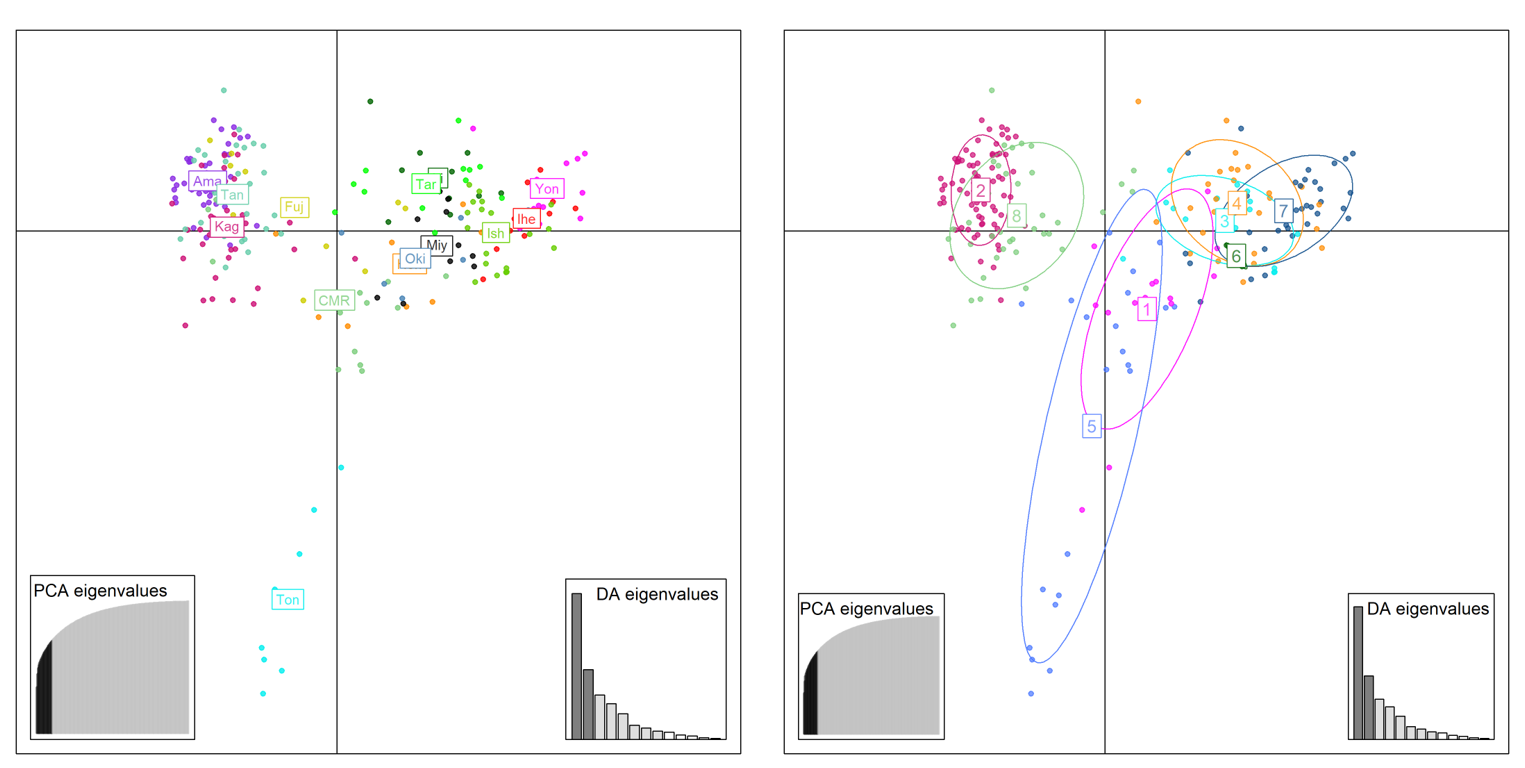


Figure S3 DAPC of Ryu+TaiA clustering. Colors indicate islands. TaiB is not considered due to strong divergence.


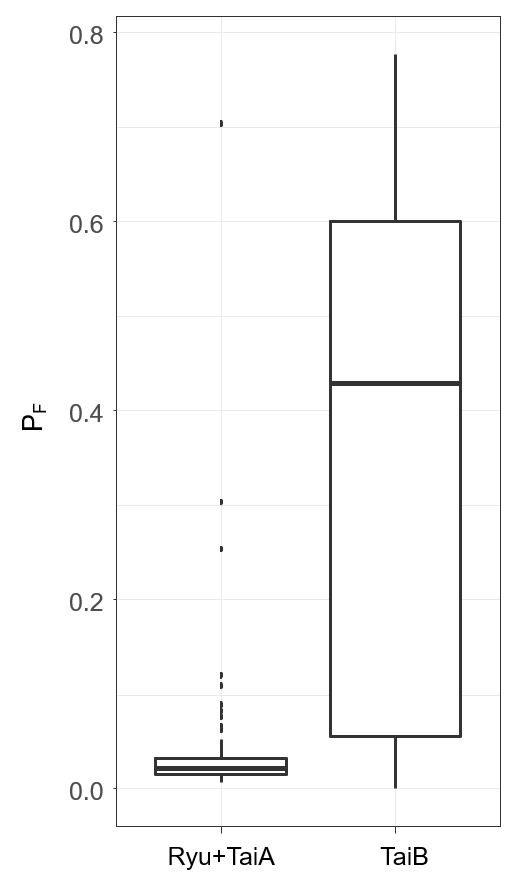


Figure S4 Private allele frequency of Ryu+TaiA and TaiB of the neutral SNPs.


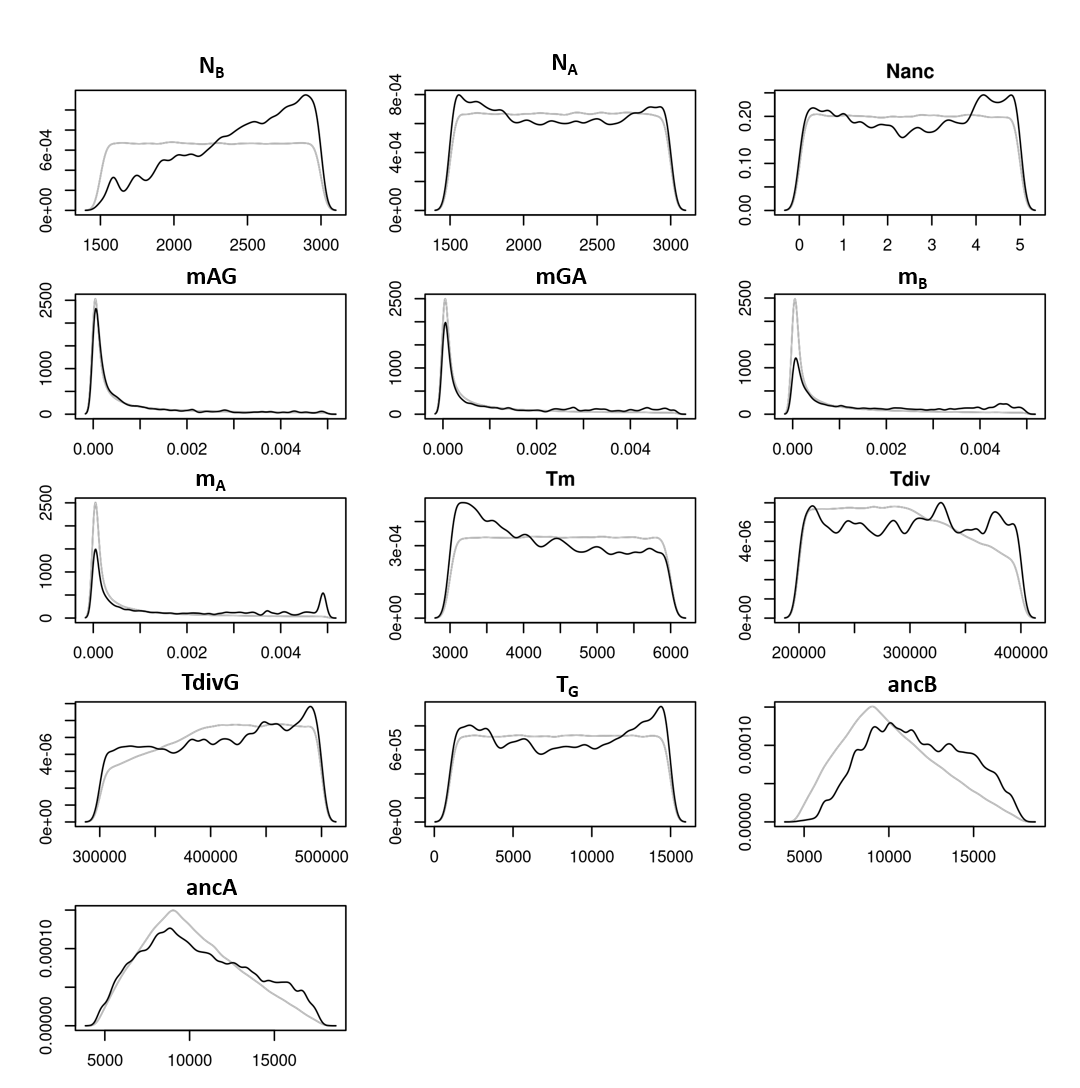


Figure S5 Posterior distribution of parameters in the best GhGeA model.

References

Beerli, P. (2004). Effect of unsampled populations on the estimation of population sizes and migration rates between sampled populations. *Molecular Ecology, 13*(4), 827-836. doi:10.1111/j.1365-294x.2004.02101.x

Chang, J. T., Chao, C. T., Nakamura, K., Liu, H. L., Luo, M. X., & Liao, P. C. (2022). Divergence With Gene Flow and Contrasting Population Size Blur the Species Boundary in Cycas Sect. Asiorientales, as Inferred From Morphology and RAD-Seq Data. *Front Plant Sci, 13*, 824158. doi:10.3389/fpls.2022.824158

Liu, J., Lindstrom, A. J., Marler, T. E., & Gong, X. (2021). Not that young: combining plastid phylogenomic, plate tectonic and fossil evidence indicates a Paleogene diversification of Cycadaceae. *Annals of Botany*. doi:10.1093/aob/mcab118

Slatkin, M. (2005). Seeing ghosts: the effect of unsampled populations on migration rates estimated for sampled populations. *Molecular Ecology, 14*(1), 67-73. doi:10.1111/j.1365-294X.2004.02393.x

Tricou, T., Tannier, E., & de Vienne, D. M. (2022a). Ghost lineages can invalidate or even reverse findings regarding gene flow. *PLoS Biology, 20*(9), e3001776. doi:10.1371/journal.pbio.3001776

Tricou, T., Tannier, E., & de Vienne, D. M. (2022b). Ghost Lineages Highly Influence the Interpretation of Introgression Tests. *Systematic Biology, 71*(5), 1147-1158. doi:10.1093/sysbio/syac011
